# Image Deblurring via Frequency-Domain Feature Enhanced Convolutional Neural Networks

**DOI:** 10.3390/s26061784

**Published:** 2026-03-12

**Authors:** Yecai Guo, Lixiang Ma, Yangyang Zhang

**Affiliations:** 1 School of Electrical and Electronic Engineering, Anhui Institute of Information Technology, Wuhu 241000, China; guo-yecai@163.com; 2 College of Electronic and Information Engineering, Nanjing University of Information Science and Technology, Nanjing 210044, China; 202412492563@nuist.edu.cn; 3 State Key Laboratory of Millimeter Waves, Nanjing 210096, China

**Keywords:** image deblurring, frequency domain, feature, Fourier transform, deep learning, spatial feature

## Abstract

To address the issues of insufficient restoration of texture details in deblurred images and inadequate learning of frequency domain features, an image deblurring algorithm based on frequency domain feature enhancement and convolutional neural networks is proposed. In this architecture, firstly, a Fourier residual module with a parallel structure is constructed to achieve collaborative learning and modeling of spatial and frequency domain features, aiming to improve frequency domain feature learning capability and the restoration effect of the texture details; secondly, a gated controlled feed-forward unit acts on the Fourier residual module to further enhance the nonlinear expression ability of the algorithm; thirdly, a supervised attention module is improved and added to the decoder to promote more effective capture of key features for image reconstruction; finally, the weighted sum of spatial domain Charbonnier loss function and frequency domain loss function is defined as a novel total loss function. In addition, to verify the performance of our proposed algorithm, we conducted experiments on the GOPRO and HIDE datasets. Through experiments on the GOPRO, we obtained an SSIM and an LPIPS of 0.961 and 0.0278, respectively. With regard to the experiments on the HIDE datasets, we obtained an SSIM and an LPIPS of 0.941 and 0.0286, respectively. As for parameter count and running time, their values were 1.197 and 9.15 × 10^6^, respectively, obtained by the experiments on the GOPRO. In all algorithms, the values of our proposed algorithm are optimal. However, the PSNR of our proposed algorithm is very close to that of the latest comparison algorithm and is suboptimal. In a word, experimental results have demonstrated that our proposed algorithm effectively removes blur while better preserving the details and edges of the image. Therefore, it has more practical value and prospects in computer vision tasks.

## 1. Introduction

A clear image preserves more information than a blurred image. However, blurred images are very common in our daily lives. Images are often blurred due to many factors such as human operation, jitter of imaging equipment, and rapid movement of objects, etc. These cause the edges of the image to become blurred and details of the image to be lost, which greatly reduces the accuracy of computer vision tasks. Therefore, image deblurring tasks need to be tackled urgently [1,2].

In the past decades, a lot of research work has been carried out by many scholars in order to obtain a clear latent image. Traditional image deblurring algorithms, such as filtering algorithms [3], the least squares algorithm [4], and equalization filters [5], etc., rely on various constrained conditions and prior knowledge. In recent years, deep learning algorithms have been widely used in the field of computer vision, such as image deraining [6,7], image denoising [8,9], image deblurring [10,11,12,13,14,15], and, recently, the particularly popular human activity recognition [16,17,18]. Learning-based algorithms have achieved good results in image deblurring tasks. Most of the existing estimation methods for learning blur kernels use Convolutional Neural Networks (CNNs) to extract blur kernels [19,20]. However, such algorithms depend excessively on the accuracy of blur kernel estimation. In the case of inaccurate blur kernel estimation, the quality of the recovered image is poor. As a result, more and more scholars have recently been devoted to the study of end-to-end image deblurring algorithms that do not completely rely on fuzzy kernel estimation [21,22,23,24,25]. To recover a latent clear image directly from a blurred image, these methods directly learn the nonlinear mapping relationship between the blurred image and the clear image instead of learning how to estimate blur kernels, which leads to the errors caused by the estimation of blur kernels being reduced. Afterwards, some scholars have further developed some deep learning-based image deblurring algorithms, such as cross-scale weight sharing algorithms [24], encoder–decoder algorithms with nested skip connections [25], multi-scale end-to-end algorithms with stacked subnetworks [23,24], and the Multi-Input Multi-Output U-shape Network (MIMO UNet) [22], etc. These algorithms can improve multi-scale feature fusion and image deblurring effect to some extent, but their performance on deblurring the image is highly dependent on high-quality blurry–clear image pairs in the dataset, and they inevitably increase computational time.

In recent years, existing image deblurring algorithms have mainly focused on feature learning in the spatial domain to estimate latent, clear images. However, in the image deblurring tasks, frequency domain feature information also plays an important role in modelling the nonlinear mapping relationship between the blurred image and the clear image. Since the low-frequency part of an image contains global information that carries the overall contour, structure and color distribution, while the high-frequency part contains edges, textures and rich local details, it is necessary to study the image blurring problems in the frequency domain. At present, some researchers have begun to introduce frequency domain feature learning methods into image deblurring algorithms [26,27], which are used to improve the texture structure and clarity of restored images. The advantage of these algorithms is that they can effectively separate frequency components and process them separately, but their ability to restore fine textures and details is still limited, and the restored image still has blurred edges and lost details.

From the above analyses, it can be seen that both traditional image-deblurring algorithms and learning-based image deblurring algorithms, as well as both spatial domain image deblurring algorithms and frequency domain image deblurring algorithms, find it difficult to achieve ideal deblurring effects, which affects the application of these algorithms in computer vision tasks.

To tackle the above problem, we propose a new image deblurring algorithm via Frequency-Domain feature Enhanced convolutional neural Network (FDENet). In our proposed FDENet, the single U-shape net is used as the backbone network, and the multi-level and multi-scale encoder and multi-level and multi-scale decoder are used. For enhancing the deblurring effect of the algorithm, we propose the frequency domain enhancement block throughout the entire deblurring process. We propose that Fast Fourier Transform Residual Block (FFT-Res) with the spatial domain branch and the frequency domain branch is used to improve the ability of the algorithm to efficiently extract texture and edge information. To improve the feature representation and the modeling ability of the algorithm, we introduce a Gated Feed-forward Unit (GFU). For conveying useful feature information within the network, we improve the existing supervised attention block and add it to the decoding modules. Finally, we propose a novel total loss function, which is the weighted sum of the spatial domain Charbonnier loss function and the frequency domain loss function, for reducing the gap between the latent clear image and the clear image in terms of spatial structure and image edges, respectively.

The contributions of our proposed FDENet can be summarized as follows:

1. We propose a new network structure for an image deblurring algorithm. It contains a multi-level and multi-scale feature encoder and a multi-level and multi-scale feature decoder.
2. To enhance the spatial and frequency feature extraction capability of the algorithm structure for image deblurring algorithm, the frequency domain enhancement block runs through the entire deblurring process, a Fast Fourier Transform Residual Block (FFT-Res) and a Gated Feed-forward Unit (GFU) are introduced to the encoder, a Supervised Attention Block (SAB) is improved and added to the decoder. This structure of our algorithm can better integrate feature information of different scales for restoring the edge and detail information of the image.
3. Finally, in order to train our algorithm to produce a latent clear image, we propose a novel total loss function that is the weighted sum of the spatial domain Charbonnier loss function and the frequency domain loss function.

## 2. Materials and Methods

### 2.1. Network Structure

**Figure 1:**
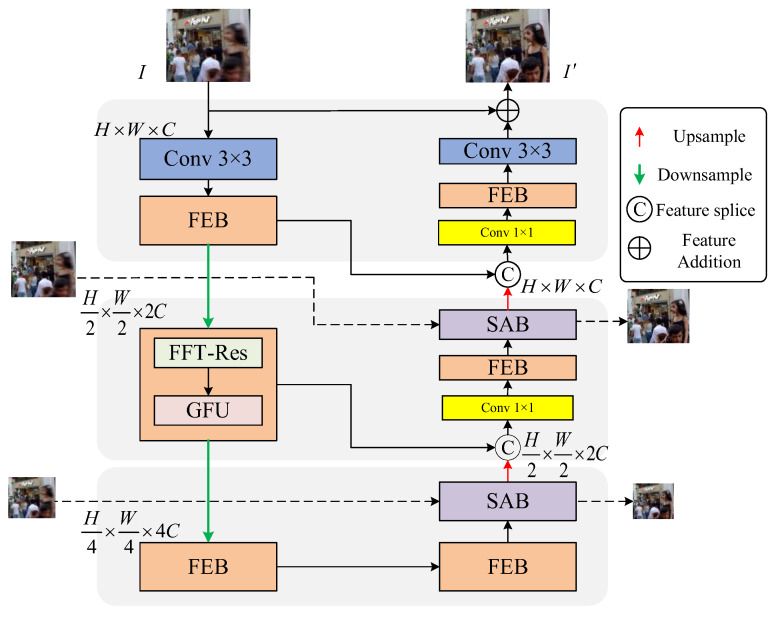
Network architecture.

The overall structure of the image deblurring algorithm via Frequency-Domain feature Enhanced convolutional neural Network (FDENet) is proposed and shown in Figure 1. The proposed FDENet adopts a single U-shape network structure to realize the information transmission between the multi-level and multi-scale encoder and the multi-level and multi-scale decoder through hopping connections. The blurred image is used as the input of the proposed FDENet, and the shallow features are firstly obtained by a 3 × 3 convolution with LeakyReLU activation function. The encoder consists of two Frequency-domain Enhancement Blocks (FEBs), a Fast Fourier Transform Residual Block (FFT-Res), and a Gated Feed-forward Unit (GFU), as well as a 3 × 3 convolution. Among them, two FEBs can improve the deblurring effect through the enhancement of the frequency domain feature information. The shallow features of the image are gradually extracted to obtain deep feature $F_{enc,i}$ through the three-level encoder. The deep feature $F_{enc,i}$ is expressed as:

(1) $$F_{enc,i}={\mathrm{Encoder}}_{i}(F_{enc,i-1})$$

where $F_{enc,i}$ denotes the output feature of the *i*th-level encoder, and ${\mathrm{Encoder}}_{i}$ stands for the composite function of the *i*th-level FEB. After the feature $F_{enc,i}$ of the *i*th-level encoder is passed to the *i* + 1 th-level encoder through downsampling, the spatial sizes *H* and *W* of the feature map are halved and the number of channels *C* is expanded by two-fold, with *H* and *W* representing the height and width of the feature map (or input image), respectively. After the feature is extracted by the encoder at each scale, the resulting feature map is passed through the corresponding decoder. The decoder consists of three FEBs, two Supervised Attention Blocks (SABs), a 3 × 3 convolution, and two 1 × 1 convolutions. The decoding process of the decoder may be divided into three stages, each of which corresponds to the three stages of the encoding process. In our proposed FDENet, we adopt an asymmetric structure, where the decoder contains an additional SAB compared to the encoder to improve the feature learning capability. After upsampling the features output by the SAB, the spatial sizes *H* and *W* of the feature map are doubled and the number of channels *C* is halved. The upsampled feature map is aggregated with the corresponding encoding stage through skip connections and expressed as follows:

(2) $$F_{dec,j}=H_{FEB}(F_{SAB,j}+F_{enc,i})$$

where $F_{dec,j}$ denotes the output feature of the *j*th-level decoder, $F_{SAB,j}$ stands for the output feature of the *j*th-level SAB in the decoding stage, and $H_{FEB}$ represents the composite function of the FEB.

In Figure 1, skip connections directly transmit features from the shallow encoding layers to the corresponding decoding layers, where they are concatenated with the decoder features and then passed through a 1 × 1 convolution to adjust the channel dimensions. This design is to ensure that the feature maps are additive and connectable and preserve the spatial details and texture information that are crucial for restoring blurred images. After the last decoder, a 3 × 3 convolution is applied to the feature map to obtain the residual image, which is added to the original image to get hold of the final restored image.

### 2.2. Fast Fourier Transform Residual Block

**Figure 2:**
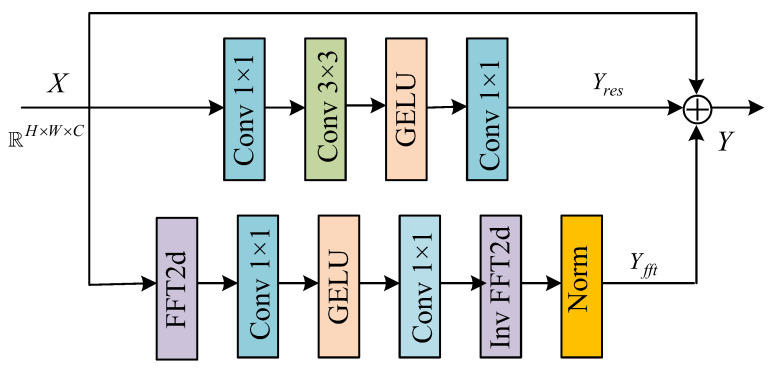
FFT-Res block.

The structure of Fast Fourier Transform Residual Block (FFT-Res) is shown in Figure 2, which consists of a spatial domain branch and a frequency domain branch. By jointly learning features in the spatial and frequency domains, the ability of the algorithm to model the details and structures of the image is effectively enhanced. In the frequency domain branch, the input feature map X undergoes a two-dimensional fast Fourier transform to obtain its imaginary part *f_i_*(*x*) and real part *f_r_*(*x*). Subsequently, these two parts are spliced in the same channel dimension. The splicing result *f* constructs the relationship between the high-frequency and low-frequency information of the image through cascaded convolutional layers, which consist of two 1 × 1 convolutions and a GELU activation function. In Figure 2, ⊕ denotes summation.

Compared to the spatial domain global feature learning method relying on deeper networks or larger receptive fields, the frequency domain feature learning method with lower structural complexity can efficiently capture the global structural information of the blurred image. At the same time, more obvious high-frequency features help the algorithm to efficiently extract texture and edge information and restore image details. So, our aim is to more effectively model the high-frequency and low-frequency feature information in the image via transferring the feature map to the frequency domain, thereby enhancing the restoration effect of texture details and overall visual quality of the deblurred image. The feature information learned in the frequency domain needs to be split into two parts to form new real and imaginary parts. Then, the Fourier inverse transform is used to transform the features from the frequency domain back to the spatial domain to obtain the output of the frequency domain branch.

In order to obtain new real and imaginary parts of the image, first, the feature information learned in the frequency domain needs to be transferred back to the spatial domain and fused with another branch to perform a splitting operation. Then, the inverse Fourier transform is used to transform the frequency domain features back to the spatial domain features to get the spatial domain output $Y_{fft}$ of the frequency domain branch.

The above computational process can be represented as:

(3) $$\;f^{\prime }=Conv(\sigma (Conv(f))$$

(4) $$\left(f_{r}^{\prime },f_{i}^{\prime })=chunk(\;f^{\prime }\right)$$

(5) $$Y_{fft}=ifft(f_{r}^{\prime }+j*f_{i}^{\prime })$$

where *fft* denotes the Fast Fourier Transform (FFT), *ifft* represents the Fourier inverse transform, *chunk* indicates a splitting operation, *Conv* stands for the 1 × 1 convolution operation, and *σ* is the ReLU activation function.

In order to reduce computational consumption that is caused by the use of the Fourier Transform (FT) in the frequency domain branch, software and hardware collaboration is required. Therefore, the FFT, residual connection, and attention mechanism are used for the computation simplification of the algorithm. In terms of implementation, it can rely on GPU and optimization libraries for acceleration; In strategy, the scaling processing is adopted.

In the FFT-Res block, the frequency domain branch maps the spatial domain features into the frequency domain features through the FFT. The output (especially amplitude components) of this branch may sometimes produce larger amplitude values than the original spatial domain features due to frequency domain enhancement operations, which may lead to gradient instability, feature distortion, or difficulty in training convergence. In order to avoid this problem or in the event of such a problem, it is necessary to ensure that the outputs of the frequency domain and spatial domain branches have comparable scales before fusion. Here, our specific approach is to normalize the outputs of the frequency domain at the channel level.

The above process can be represented as:

(6) $$Y=Y_{fft}+Y_{res}+X$$

### 2.3. Gated Feed-Forward Unit

**Figure 3:**
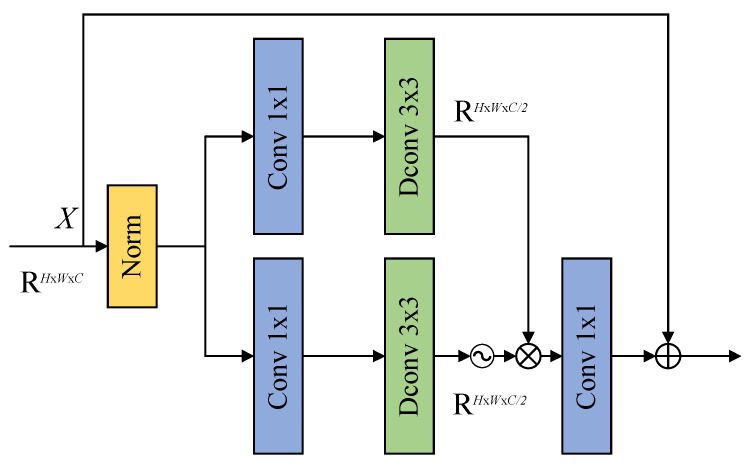
Gated Feed-forward Unit (GFU).

In order to improve the feature representation capability and the ability to create the algorithm model, a Gated Feed-forward Unit (GFU) is cascaded after the FFT-Res block, as shown in Figure 3. The feature information extracted by the FFT-Res block contains both spatial and frequency domain information. So, it can adaptively enhance important feature information and suppress redundant feature information through a gating mechanism, thereby improving the deblurring effect. The working process of the GFU is as follows:

Firstly, the input features X are subjected to layer-normalization to stabilize the training process and speed up the convergence of the model.

Secondly, the layer-normalized features are divided into upper and lower two branches based on the number of channels, and then the number of channels is doubled through a 1 × 1 convolution. Afterwards, the local spatial modelling capability is introduced through a 3 × 3 depth-wise separable convolution. In two branches, after the lower branch is activated by the GELU function to form a gating unit, it is multiplied element-by-element with the features of the upper branch to achieve adaptive modulation of the feature information. The gating mechanism can strengthen the important feature information and suppress the redundant features, thus effectively improving the expression ability of the features. In Figure 3, 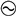 denotes GELU activation function, ⊗ denotes element-by-element multiplication operation and ⊕ denotes summation.

Thirdly, the number of channels is changed to the initial dimension through a 1 × 1 convolution. Based on combining the depth-wise convolution with the gating mechanism, we use the GFU to improve the ability of the network to map the nonlinear feature and extract the features from the FFT-Res block. The mathematical expression for the GFU is written as:

(7) GFU=Conv(Gating(X))+X

(8) X=σ(DConv(Conv(LN(X))))⊗DConv(Conv(LN(X)))

where ⊗ denotes element-by-element multiplication operation, *Conv* represents a 1 × 1 convolution operation, *DConv* stands for depth-wise convolution operation, *LN* signifies layer-normalization operation, and *σ* is the GELU activation function.

### 2.4. Supervised Attention Block

**Figure 4:**
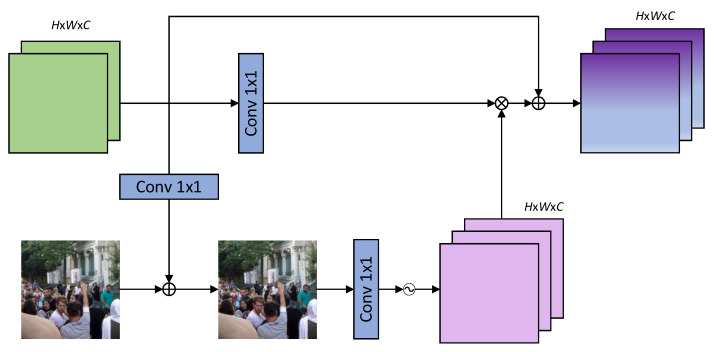
Supervised attention block.

Recently, Supervised Attention Blocks (SABs) have achieved good results in multi-stage image recovery tasks. Therefore, we introduce the SABs to our algorithm for obtaining important feature information. The downsampled blurry images are directly passed to the next stage, but the feature information passed to the next stage may not be useful for deblurring the image. Therefore, in order to convey useful and abundant feature information as much as possible, the SAB module is added between two consecutive decoders during the upsampling process to provide ground-truth supervision in the training stage of the algorithm, which is used to filter out useless feature information and train a better algorithm model. The SAB has two functions: (1) providing the real image supervision signals during training, which helps to deblur the images in the next stage; (2) the generated attention features aim to suppress the transmission of useless features to the next stage. The SAB module is shown in Figure 4; its input comes from the output features of the previous stage for deblurring. In Figure 4, 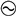 denotes GELU activation function, ⊗ denotes element-by-element multiplication operation and ⊕ denotes summation.

According to Figure 4, firstly, the input from the output features of the previous stage X is convolved through 1 × 1 convolution to get residual features, which are added to the low-scale blurred image I to generate a low-scale predicted image $I^{\prime }$. This low-scale predicted image is used as a ground-truth supervised image. And this is helpful in reducing the pixel differences between the final predicted image and the ground-truth image. When the obtained predicted image is passed through a 1 × 1 convolution and the Sigmoid activation function in sequence, an attention feature map with weights is generated and multiplied element-wise with the features from another branch. Then the output of the SAB is obtained through a residual connection, which is upsampled and used as the feature input for the next-level decoder. The specific process is represented as follows:

(9) $$I^{\prime }=Conv(X)$$

(10) $$SAB(X)=Conv(Sigmoid(I^{\prime }))⊗Conv(X)+X$$

where *Conv* is a 1 × 1 convolution operation, ⊗ denotes element-by-element multiplication operation, and *Sigmoid* stands for the activation function.

A common challenge with supervised attention mechanisms is the risk of overfitting, where the module becomes overly reliant on the predicted guidance maps, leading to overfitting on training samples. To address this, the proposed SAB incorporates three key strategies to regularize the learning process. First, the SAB computes a residual adjustment to the downsampled original image. By anchoring the attention map to the underlying physical structure of the scene, the module is forced to learn more generalized image-to-feature correlations. Second, the attention weights are derived from this reconstructed image rather than abstract high-level features, ensuring that the feature gating is physically grounded. Finally, the SAB adopts a residual connection that adds the gated features back to the original input. This design ensures that the primary feature flow is preserved even if the attention guidance is suboptimal, thereby enhancing the model’s robustness and preventing it from collapsing into a biased state during training.

**Figure 5:**
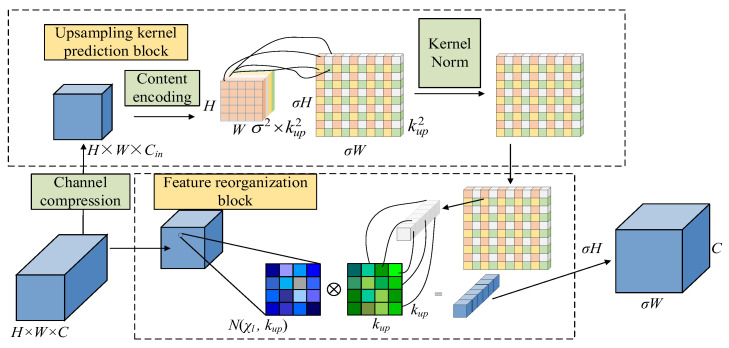
CARAFE architecture.

It should be noted that in the U-Net architecture, the skip connection from the encoder to the decoder does require aligning the spatial dimension before applying supervision or performing feature fusion, which is a key prerequisite for ensuring effective information transmission. Within the proposed architecture, each SAB receives two inputs for guided learning. One is the original clear image downsampled to match the spatial scale of the current decoder-side FEB. The other input is the feature map from the FEB, which maintains the same dimensions as its input. To facilitate feature fusion, the output from the previous stage is concatenated with the skip-connection features, followed by a 1 × 1 convolutional layer for channel recalibration. This refined feature map then passes through the FEB to match the SAB’s input configuration. In order to ensure the correct fusion of encoding and decoding feature maps during skip connections, it is necessary to achieve precise spatial and channel alignment. For achieving precise spatial dimension alignment, upsampling needs to be achieved using some methods, such as transpose convolution or bilinear interpolation. However, there are certain limitations in practical applications, such as the risk of artifacts, insufficient detail recovery, or structural distortion. Therefore, we use CARAFE (Content Aware ReAssembly of FEatures) [28] to better preserve semantic continuity and obtain higher quality feature map recovery results instead of the traditional upsampling method. The structure of the CARAFE is shown in Figure 5. During upsampling, the CARAFE adaptively generates reconstruction weights through incorporating content information from the current feature map to achieve more refined feature reconstruction. This module primarily consists of two parts: the kernel prediction module and the feature reorganization module. Among them, the upsampling kernel prediction module, used to generate upsampling kernels, comprises three sub-modules: Channel compression, content encoding, and kernel normalization. Given an input feature map of size H × W × C and an upsampling rate σ, the channels of the input feature map are first reduced using a 1 × 1 convolution to decrease subsequent computational complexity. The content encoding involves predicting upsampling kernels for the compressed feature map using a convolutional layer. The convolved feature map is then expanded along the channel dimension to obtain a recombined upsampling kernel of size σ*H* × σ*W* × *k*_up_ × *k*_up_. The Kernel normalization involves normalizing the upsampling kernel using the Softmax function before applying the feature reorganization module. The feature reorganization module is responsible for mapping each position of the output feature map back to the input feature map and performing a dot product operation between the *k_up_ × k_up_* region centered on the output feature map and the upsampling kernel of the prediction points to obtain the output. The content-aware reorganization module allows more attention to be paid to the relevant feature information in the local region, so richer feature information can be extracted by using the CARAFE as the upsampling method.

The method of achieving channel dimension alignment is to use 1 × 1 convolution to adjust the number of channels in the encoder feature map for cases where the number of channels is different, so as to match the number of input channels in the decoder and avoid channel explosion after concatenation.

### 2.5. Loss Function

In order to obtain better image deblurring effects than single spatial domain optimization, we define a novel total loss function $L_{total}$ as the weighted sum of the spatial domain Charbonnier loss function $L_{\mathrm{charbonnier}}$ and the frequency domain loss function $L_{\mathrm{freq}}$, i.e.,

(11) $$L_{total}=L_{\mathrm{charbonnier}}+\alpha \times L_{\mathrm{freq}}$$

where $L_{\mathrm{charbonnier}}$ represents the spatial domain Charbonnier loss function; $L_{\mathrm{freq}}$ denotes frequency domain loss function; α is a hyperparameter, usually determined experimentally. When α is set to 0.1 through our experiments, the obtained image has the best deblurring effect.

The core idea of Equation (11) is to simultaneously optimize the errors of the image in two different but complementary representation spaces, the spatial domain and the frequency domain. The spatial domain loss function directly measures the point-by-point difference between the predicted image and the real image on the time axis, and is sensitive to local details and waveform shapes. The frequency domain loss function measures the differences in frequency components of an image and is more sensitive to global structure, timbre, and artifacts (such as checkerboard effects). The focus of the two is different, and the weighted summation achieves a “dual approach” to simultaneously improve the structure (spatial domain) and detail texture (frequency domain) of the image.

(1) Spatial domain Charbonnier loss function

We chose the Charbonnier loss function [29] as the spatial domain loss function. The expression of the Charbonnier loss function is defined as follows:

(12) $$L_{\mathrm{charbonnier}}=\sqrt{{\left(I_{\mathrm{pred}}-I_{\mathrm{gt}}\right)}^{2}+\eta^{2}}$$

where $I_{\mathrm{pred}}$ and $I_{\mathrm{gt}}$ are the predicted image and the clear image, respectively. The square root term in Equation (12) ensures that the loss function is differentiable and smooth. Equation (12) can be interpreted as the Euclidean distance between the predicted image and the clear image and has the added benefit of being less sensitive to outliers due to the square root term. This property makes it particularly useful in many tasks, such as image denoising, optical flow estimation, and depth map estimation, where robustness to outliers is important. One of the advantages of the Charbonnier loss function is that it is differentiable everywhere, except at $I_{\mathrm{pred}}$ = $I_{\mathrm{gt}}$. Furthermore, the Charbonnier loss function helps address the challenges of precise image reconstruction and alignment via balancing accuracy and robustness.

Compared to the L1 loss function [30], the presence of a constant term *η* in Equation (12) ensures that the gradient of Equation (12) is not too small when the loss function approaches zero, and here we take *η* as 0.001.

(2) Frequency domain loss function

The frequency loss [29] function in Equation (12) is regarded as an auxiliary term in the total loss function, usually referring to a type of function that calculates the loss by comparing the difference between the predicted image and the real image in the frequency domain. Here, the expression of the frequency domain loss function is defined as follows:

(13) $$L_{freq}=\sqrt{{\left(F(I_{\mathrm{pred}})-F(I_{\mathrm{gt}})\right)}^{2}+\eta^{2}}$$

where F denotes the FFT, and the meaning of η is the same as Equation (11).

Since the details, textures, and other information of an image are often reflected in high-frequency components, while the overall loss function is mainly reflected in low-frequency components, it is an innovative approach for us to use Equation (11) to balance spatial and frequency domain features to achieve better image deblurring effects and restore visual quality.

## 3. Results

### 3.1. Dataset and Parameter Settings

The dataset used in this section is derived from the video sequences captured by Zhang et al. [31] using GoPro cameras, and the blurred images are obtained by averaging consecutive exposure frames. The GoPro dataset consists of 3214 pairs of blurred and clear images at a resolution of 1280 × 720, 2103 pairs of which are used for the training set and 1111 pairs of which are used for the testing set. Additionally, to analyze the generalization ability of the algorithm, the HIDE dataset proposed by Shen et al. [32] is used for comparative experiments, which covers a wide range of scenes and various types of non-uniform blurring. The HIDE dataset consists of 8422 pairs of blurred and clear images, 2025 pairs of which are employed in the testing set.

The experiments were conducted on the Windows 11 OS, with a Python 3.6 compilation environment and PyTorch 1.7.1 as the deep learning framework. All results were obtained by running the experiment on an NVIDIA GeForce RTX 3060 Ti. During data preprocessing, the input images were first randomly cropped to 256 × 256 pixels, and then horizontally flipped with a 50% probability to enhance data diversity and the generalization capabilities of the algorithm. The experiments were trained for a total of 3000 epochs, with a batch size of 4. The Adam optimizer with a warm-up strategy was used for optimization, where the momentum decay exponents of the Adam optimizer were set to $\beta_{1}=0.9$ and $\beta_{2}=0.999$. The learning rate adjustment strategy employed a more robust cosine annealing strategy, with an initial learning rate of 1 × 10^−4^ and a minimum learning rate of 1 × 10^−6^.

### 3.2. Quantitative Comparative Analyses

To validate the effectiveness of each module of the design, quantitative comparison experiments were conducted in this section. Peak Signal-to-Noise Ratio (PSNR) and Structured Similarity Index Measurement (SSIM) [33] were used as the quantitative evaluation of the image quality. The higher the PSNR and SSIM values of the algorithm, the better its performance in deblurring capabilities and reconstructing the quality of the image. Therefore, the performance of our proposed FDENet was evaluated on the benchmark datasets GoPro and HIDE, and compared with the SRN [23], PSS-NSC [24], DMPHN [31], SDWNet [27], VDN [34], MSAN [35], BANet [36], MPRNet [37], EHNet [38] and MIMO-UNet [22], as shown in Table 1.

As shown in Table 1, on the GoPro dataset, our proposed FDENet achieved a PSNR value of 32.85 dB, whereas the SSIM value reached 0.961 and was second only to that of the SDWNet, which used wavelet transformation and achieved the second-best value. Compared to the MPRNet, which stacks multiple subnetworks, the PSNR of our FDENet has an improvement of about 0.19 dB, and the SSIM has an improvement of about 0.02. On the HIDE dataset, our FDENet achieved the PSNR value of 30.93 dB and the SSIM value of 0.941. The PSNR of our FDENet has a drop of about 0.03 dB and is second only to that of the MPRNet, whereas the SSIM has an improvement of about 0.02 and belongs to the optimal value. While EHNet achieves marginally higher PSNR on both datasets due to its transformer-based architecture with greater computational demands, our FDENet demonstrates a clear advantage in runtime efficiency, as evidenced in the subsequent comparison in Section 3.5. These experimental results have demonstrated overall excellent performance of our FDENet in deblurring the image and improving the ability to effectively fuse spatial and frequency domain features, utilizing global information through the frequency domain enhancement block and alleviating the limitations of transposed convolutions during upsampling. The experimental results also indicate that our FDENet has good generalization capability in complex blurring scenarios.

### 3.3. Comparative Analyses Combining Qualitative and Quantitative Methods

(1) Benchmark dataset

**Figure 6:**
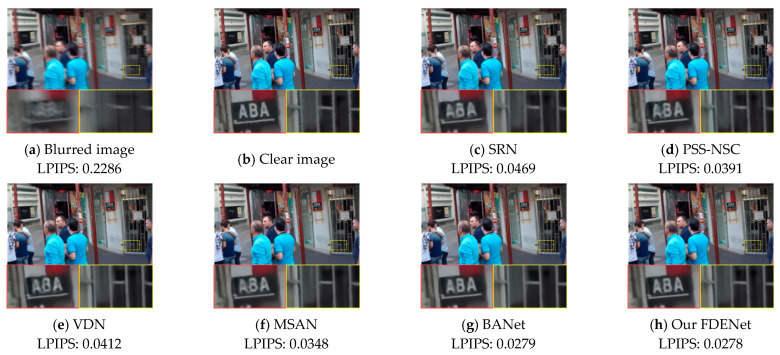
Visual comparison of different algorithms on the GoPro dataset.

**Figure 7:**
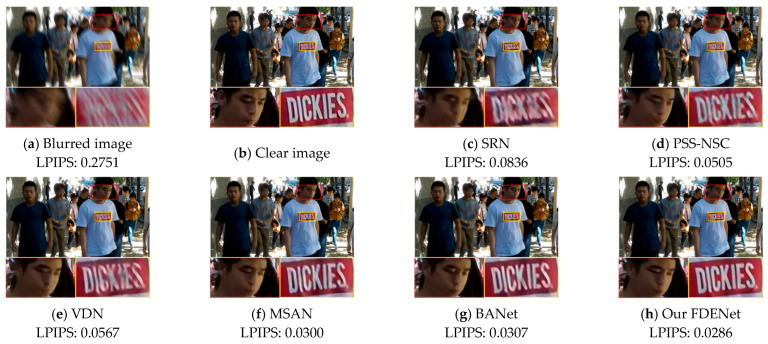
Visual comparison of different algorithms on HIDE dataset.

To demonstrate the effectiveness of our FDENet, qualitative analyses were conducted by comparing it with existing mainstream image deblurring algorithms. Figure 6 and Figure 7 visually present the comparative visual effects of the SRN, PSS-NSC, VDN, MSAN, BANet, and FDENet on the GoPro dataset and the HIDE dataset, respectively. To comprehensively evaluate human-perceived quality and other complex factors, we introduce LPIPS to objectively assess the capabilities of these algorithms.

In Figure 6, residual blurring still exists after processing using the SRN and MSAN, which results in a certain degree of deformation in the letter structure. The image processed by the BANet appears overly smooth in the edge areas of the letters. In contrast, our proposed FDENet performs better in restoring clear edges and fine details, achieving the best LPIPS score of 0.0278. In Figure 7, the SRN still exhibits severe blurring artifacts and is unable to recognize specific textual information. The image processed by the PSSNSC also suffers from color distortion. Our proposed FDENet restores letters and faces more clearly without severe artifacts, resulting in outputs that are closer to the real, clear image. Compared with other algorithms, it still achieves the best LPIPS. Through frequency domain feature enhancement, our proposed FDENet effectively removes blurring while preserving the texture and structure of the original image, making the deblurred image more natural and realistic.

(2) Actual shot motion-blurred image

**Figure 8:**
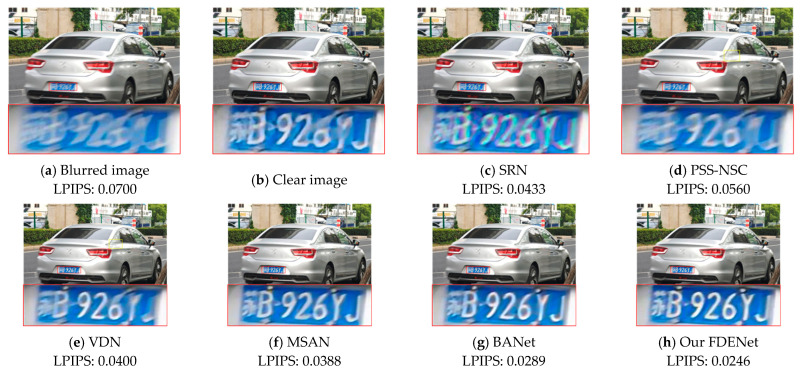
Visual comparison of different algorithms on real blurred images.

The blurred image in Figure 8 was captured by a smartphone with a fixed camera position while a car was moving rapidly. Such blurs are extremely common in real-life scenarios. To verify the deblurring performance on real-world blurred images, qualitative comparative experiments were conducted between our proposed FDENet and other algorithms. As can be seen from Figure 8, the SRN and VDN exhibit poor deblurring effects in practical applications, and the restored images remain very blurry. The PSS-NSC and DMPHN improve the deblurring effects to a certain extent, but they still fail to effectively restore image details—the license plate in Figure 8 stays blurry. In contrast, our proposed FDENet effectively restores blurry images caused by rapid object movement, yielding clearer details and a more realistic effect. Quantitatively, our FDENet achieves the best LPIPS score of 0.0246 among all compared algorithms, further demonstrating its superior perceptual quality.

### 3.4. Impact on Subsequent Visual Tasks

**Figure 9:**
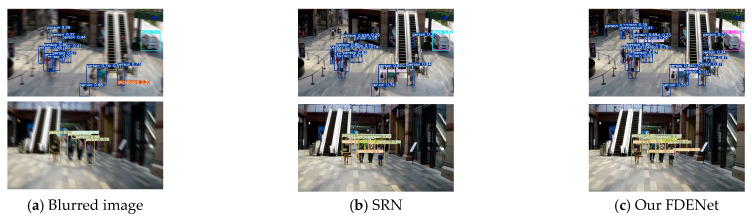
Comparison of results of target detection.

Image deblurring, as a low-level visual task, can be applied to the preprocessing stage of advanced computer vision tasks, such as image classification, recognition, and object detection. When the input to a detection algorithm is a blurry image, it can lead to false positives and false negatives, thereby affecting detection accuracy. In this section, to verify the effectiveness and feasibility of our proposed FDENet in processing the blurred images, we conducted verification experiments based on the YOLOv7 object detection algorithm. The visualization results of object detection for the blurred images, the images processed by the SRN, and those processed by our FDENet are presented in Figure 9. It can be seen that the blurry images severely compromise detection effectiveness, with obvious missed detections. Quantitatively, the recall and F1 score for the blurry images are 48.15% and 65%, respectively. After deblurring with the SRN, the recall improves to 55.56%, and the F1 score to 71.43%, yet some missed detections remain. In contrast, our FDENet restores texture and edge details more effectively, producing no false positives and achieving the best performance: A recall of 85.19% and an F1 score of 92%. These results demonstrate that our FDENet, through frequency domain feature enhancement, not only removes blur but also improves the performance and robustness of subsequent visual tasks.

### 3.5. Runtime and Parameter Analyses

The running time and parameter counts of our FDENet and other comparative algorithms on the GoPro testing set with a resolution of 1280 × 720 pixels are shown in Table 2. From Table 2, we can know that the parameter count of the SRN is 6.8 × 10^6^ with a runtime of 0.814 s, the parameter count of the MPRNet is 20.1 × 10^6^ with a processing time of 1.002 s, the parameter count of the EHNet is 8.77 × 10^6^ with a processing time of 1.197 s, and the parameter count of our FDENet is 9.15 × 10^6^ with a running time of 0.347 s. Furthermore, among all algorithms, although the EHNet achieves excellent performance by using the transformer method, it also takes the longest running time. So, our FDENet maintains the highest speed while having a relatively small parameter count, thereby resulting in the best real-time performance.

### 3.6. Ablation Experiment

To verify the effectiveness of the upsampling module in our FDENet, the CARAFE was replaced with the commonly used transposed convolution and bilinear interpolation, respectively. The comparative results are shown in Table 3. When using the CARAFE as the upsampling module, the PSNR has an improvement of about 0.14 dB compared to the transposed convolution. Compared to the bilinear interpolation method, the PSNR also has an improvement of about 0.08 dB. Additionally, the CARAFE introduces only a small number of parameters and computational costs. Therefore, the CARAFE is selected as the upsampling method in our FDENet.

**Figure 10:**
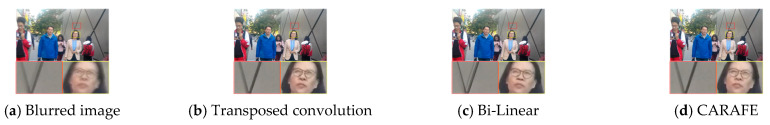
Visual comparison of different up-sampling methods.

Figure 10 shows a comparison of the visual effects of three upsampling methods, namely transposed convolution, bilinear interpolation, and the CARAFE, in image deblurring tasks. All three methods demonstrate excellent overall deblurring effects, but there are significant differences in their handling of the details. The CARAFE can enhance feature learning capabilities by expanding the local receptive fields, so it will result in clearer detail restoration and can effectively reduce the generation of artifacts.

Table 4 presents the ablation experiment results of each module in our FDENet on the GoPro testing dataset. As shown in Table 4, introducing GFU alone does not significantly improve the performance of the algorithm, with only an improvement of about 0.04 dB for the PSNR. This indicates a weak enhancement effect on deblurring performance in the absence of other structural synergies. When the FFT-Res and GFU work together, the PSNR has an improvement of about 0.83 dB compared with the ordinary residual. The experimental results have verified the effectiveness of each module in improving deblurring performance. Furthermore, on the condition of the synergistic effect of various modules, the ability of our FDENet to deblur under complex dynamic blur scenarios is further improved.

**Figure 11:**
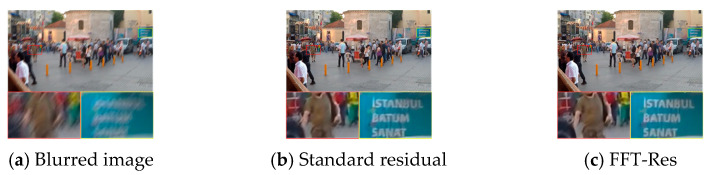
Visual comparison of different residual methods.

To intuitively validate the effectiveness of the FFT-Res in our FDENet, it is visually compared with the network using a standard residual structure. As shown in Figure 11, the network with the FFT-Res can achieve better deblurring effects for text edges and structural components in the processed images. By enhancing the ability of the network to learn frequency domain features, it can further improve its capacity to represent global image information and high/low-frequency feature information, thereby demonstrating superior performance in texture detail restoration.

### 3.7. Module Complexity Analyses

To analyze the computational complexity of each module in our FDENet under various hardware configurations, we provide the parameter counts, floating-point operations (FLOPS), and runtime for each module in Table 5. This facilitates an understanding of the resource consumption and significance of the different components within our FDENet. It should be noted that the input dimensions for the FFT-Res and GFU modules are consistent with those of the first-layer encoder, while the SAB module aligns with the second-layer decoder.

As illustrated in Table 5, there exists a distinct trade-off between computational overhead and functional efficacy among the modules. The FFT-Res module exhibits the highest parameter count (24.7 k) and computational cost (1082.2 M FLOPS), underscoring its role as the primary feature extractor in the frequency domain, which is fundamental to the model’s performance. In contrast, the GFU module, despite having fewer parameters (5.5 k), records the longest runtime (1.390 ms). This suggests that its efficiency is less dependent on parameter scale but rather on the complex memory access patterns or non-linear operations inherent in gating mechanisms. The SAB module proves to be the most lightweight, with the fewest parameters (4.5 k) and fastest inference (0.209 ms), validating its design as an efficient plug-and-play component for feature refinement at the decoder stage. This analysis confirms that while FFT-Res provides the foundational representations, GFU introduces a critical, albeit computationally intensive, gating function, and the SAB offers efficient fine-tuning with minimal resource consumption.

## 4. Discussion

According to the above experiments and the improved performance, the novelty and contribution of our proposed FDENet can be shown.

First, the algorithm tries to tackle the issue of image deblurring. We construct a new algorithm that adopts the single U-shape network structure to realize the information transmission between the encoder and the decoder through hopping connections. Frequency domain Enhancement Block (FEB) is designed in an encoder–decoder to realize the learning of spatial and frequency domain feature information. A Fast Fourier Transform Residual Block (FFT-Res) with the spatial domain branch and the frequency domain branch improves the ability of the algorithm to efficiently extract and restore texture detail and edge information. In order to enhance the feature representation and modeling ability of the network, a Gated Feed-forward Unit (GFU) is cascaded after the FFT-Res module in the encoding stage. It uses a normalization operation to stabilize the training process and accelerate the convergence speed. The element-wise multiplication is used to achieve adaptive modulation of the feature information, which can adaptively enhance important feature information, suppress redundant feature information, and effectively improve the representation ability of the image features and the deblurring effect. We improve and add the Supervised Attention Block (SAB) module in the decoding stage. This module uses the CARAFE instead of traditional upsampling methods to better preserve semantic continuity and acquire effective information for image deblurring, thus enhancing the feature extraction capability of the network. Finally, we propose a novel total loss function that is the weighted sum of the spatial-domain Charbonnier loss function and the frequency domain loss function for reducing the gap between latent clear images and clear images in terms of spatial structure and image edges, respectively.

Second, a large number of experiments have been conducted. The experimental results according to Section 3.2 and Section 3.3 show that our proposed FDENet outperforms existing methods in deblurring the image. In Section 3.4, we apply our proposed FDENet to the preprocessing stage of advanced computer vision tasks, conduct verification experiments based on the YOLOv7 object detection algorithm, and we find that our proposed FDENet can effectively improve the performance and robustness of high-level computer vision tasks through the frequency domain feature enhancement block. Runtime and parameter count on the GoPro datasets are compared by Section 3.5, where the experimental results show that our FDENet maintains a good running speed while having a relatively small number of parameters. To verify the effectiveness of upsampling method based on the CARAFE in our proposed FDENet, the ablation experiments with different upsampling methods and the proposed modules are carried out by Section 3.6, where the experimental results show that the CARAFE can provide clearer detail recovery and effectively reduce the generation of artifacts and the proposed each module plays an important role in improving deblurring performance of the image. In Section 3.7, we conducted module complexity analyses, whose results indicate that there exists a distinct trade-off between computational overhead and functional efficacy among the modules in our FDENet. The experimental results also show that our proposed FDENet can further enhance the recovery ability of the image in complex, dynamic, blurry scenes through the collaborative effect of each module.

In the future, we will focus on researching more lightweight and efficient networks and applying them to real-life scenarios after being validated by a more diverse set of real-world datasets. In addition, dynamic weighting of the total loss function is also one of our future research directions.

## 5. Conclusions

In this paper, a novel algorithm, FDENet, is proposed for removing blur from images. In our proposed FDENet, the Frequency domain Enhancement Block (FEB) runs through the entire deblurring process of the image to realize the learning of spatial and frequency domain feature information, the frequency domain feature is captured by the Fast Fourier Transform Residual Block (FFT-Res), the Supervised Attention Block (SAB) is improved and added to enhance the feature extraction capability of the encoder, the Gated Feed-forward Unit (GFU) is responsible for the enhancement of the feature mapping capability and implements effective propagation within the network. The experiment results have demonstrated that our proposed FDENet outperforms current image deblurring algorithms in improving the quality of the blurred images, robustness and generalization capabilities. Therefore, our proposed FDENet is suitable for computer visual tasks under different scenarios.

## Figures and Tables

**Table 1:** Test results on GoPro and HIDE datasets.

Model	GOPRO		HIDE	
	PSNR	SSIM	PSNR	SSIM
SRN	30.26	0.934	28.36	0.903
PSS-NSC	30.92	0.942	29.10	0.913
DMPHN	31.20	0.940	29.10	0.917
SDWNet	31.26	0.966	28.99	0.957
VDN	31.65	0.951	29.27	0.923
MIMO-UNet	31.73	0.951	29.28	0.920
MSAN	32.24	0.956	30.04	0.935
BANet	32.54	0.957	30.16	0.930
MPRNet	32.66	0.959	30.96	0.939
EHNet	32.99	0.961	31.19	0.941
FDENet (Our)	32.85	0.961	30.93	0.941

**Table 2:** Runtime and parameter comparison.

Model	Runtime/s	Params
SRN	0.814	6.8 × 10^6^
DMPHN	0.424	21.7 × 10^6^
MSAN	0.419	35.9 × 10^6^
MPRNet	1.002	20.1 × 10^6^
EHNet	1.197	8.77 × 10^6^
FDENet (Our)	0.347	9.15 × 10^6^

**Table 3:** Ablation experiments with different upsampling methods.

Upsampling Method	PSNR	SSIM
Transposed convolution	32.71	0.958
Bi-linear	32.77	0.959
CARAFE	32.85	0.961

**Table 4:** Results of ablation experiments.

FFT-Res	GFU	SAB	PSNR	SSIM
×	×	×	31.78	0.951
×	√	×	31.82	0.953
√	×	×	32.49	0.955
√	√	×	32.61	0.956
√	×	√	32.74	0.958
√	√	√	32.85	0.961

**Table 5:** Computational complexity of each module.

Module	Params	FLOPS	Runtime
FFT-Res	24.7 k	1082.2 M	1.080 ms
GFU	5.5 k	358.6 M	1.390 ms
SAB	4.5 k	73.4 M	0.209 ms

## Data Availability

The original contributions presented in this study are included in the article. Further inquiries can be directed to the corresponding author.
